# Consistent Condom Use and Associated Factors among HIV-Positive Clients on Antiretroviral Therapy in North West Ethiopian Health Center, 2016 GC

**DOI:** 10.1155/2019/7134908

**Published:** 2019-03-17

**Authors:** Mohammed Seid Ali, Eleni Tesfaye Tegegne, Mekibib Kassa Tesemma, Kaleab Tesfaye Tegegne

**Affiliations:** ^1^School of Nursing, College of Medicine and Health Sciences, Gondar University, Gondar, Ethiopia; ^2^Institute of Tropical Medicine (ITM), in Collaboration with University of Gondar, Gondar, Ethiopia; ^3^Department of Public Health, Hawassa Health Science College, Hawassa, Ethiopia

## Abstract

**Background:**

The burden of Human Immune Deficiency Virus or Acquired Immune Deficiency Syndrome is high in sub-Saharan countries including Ethiopia which have over two-thirds of the global HIV burden. Many would argue that consistent condom use is not most effective method for HIV prevention. Condoms offer protection against unwanted pregnancy and some sexually transmitted infections including Human Immune Deficiency Virus, when used correctly and consistently. Inconsistent use of condom by People Living with Human Immune Deficiency Virus or Acquired Immune Deficiency Syndrome on Antiretroviral Therapy will lead to further worsening the Human Immune Deficiency Virus infection epidemic and reinfection with new drug resistant viral strains.

**Objective:**

To assess magnitude of consistent condom use and associated factors among HIV-positive clients on Antiretroviral Therapy in North West Ethiopian health center, 2016 GC.

**Method:**

An institutional based cross-sectional study was conducted, from April 15 to June 10, 2016. A total of 358 patients on ART in Koladiba Health Center had participated in this research. Koladiba Health Center is the first health center in Ethiopia that is found in Debbie district, which is located in north Gondar Zone. Study participants were selected by simple random sampling technique. Data were collected by using pretested structured questionnaires and analyzed using SPSS version 22. Descriptive statistics was computed and binary and multiple logistic regressions were also conducted to examine the effect of selected independent variables on consistent condom use.

**Result:**

A total of 358 ART clients participated in the study with response rate of 90%. Among study participants, 138 (38.5%) were in the age category of 35-44 years. About 216 (60.3%) of the participants were female and 325 (90.8%) were Orthodox followers. Consistent condom use was reported by 130 (55.8%) sexually active study subjects. Respondents in rural residence (AOR=0.326, 95% CI: 0.109, 0.973) and sexual partner initiated condom use (AOR=0.031, 95% CI: 0.005, 0.186) were found to be the independent predictors of consistent condom use.

**Conclusion and Recommendations:**

Consistent condom utilization among HIV clients on ART was low (55.8%). Place of residence and condom use initiation during sexual contact were significantly associated with consistent condom use. It is better to give more emphasis on health education and counseling service about consistent condom use for PLWHA who are on ART during follow-up especially for those who came from rural areas.

## 1. Introduction

### 1.1. Statement of the Problem

Since the beginning of the epidemic, almost 71 million people have been infected with the HIV virus and about 34 million people have died of HIV. According to the World Health Organization (WHO), there were approximately 36.9 million people worldwide living with HIV/AIDS at the end of 2014. Sub-Saharan Africa remains most severely affected, with nearly 1 in every 20 adults living with HIV and accounting for nearly 70% of the people living with HIV worldwide [1]. The vast majority of people living with HIV are in low- and middle-income countries including Ethiopia [2].

Prevention methods, particularly consistent condom use, have helped to reduce HIV transmission and curtailed the broader spread of HIV in settings where the epidemic is concentrated in specific populations [3].

In Ethiopia the response to HIV/AIDS epidemic showed considerable progress and achieved encouraging results. However, the nature of epidemic and its fueling factors creates a complex challenge to the ability of health and other sectors to meet the target for HIV/AIDS prevention and control in the country [4].

Combination of HIV prevention is likely to be most effective when different points in the “transmission cycle” are impeded, combining strategies to reduce infectiousness of HIV-positive persons with strategies that reduce HIV susceptibility in the uninfected person. Most early HIV prevention policies focused heavily on HIV-negative, at-risk persons (e.g., using behavior change communication campaigns). However, seronegative persons represent a very large pool to target high coverage. Strategies to reduce the infectiousness of HIV-positive individuals by reducing secondary HIV transmission should be part of the prevention policy. Theoretically, if high proportions of people living with HIV/AIDS (PLHIV) learned their HIV serostatus and adopt interventions such as ART coupled with behavioral risk reduction, this could have a significant impact on HIV transmission [5].

Condom use is a critical component in a comprehensive and sustainable approach to the prevention of HIV and other sexually transmitted infections (STIs) and for preventing unintended pregnancies especially among people with HIV-positive clients. The maximum protective effect of condoms is achieved when their use is consistent rather than occasional [6]. Previously, the focus of HIV prevention efforts in most countries including Ethiopia was largely on people uninfected with HIV. Sexual risk practice of HIV infected persons did not receive due attention. Inconsistent use/nonuse of condom by PLWHA on ART will lead to further worsening the HIV infection epidemic and reinfection with new drug resistant viral strains. Therefore the aim of this study is to determine the level of consistent condom use and some associated factors among HIV-positive clients on antiviral therapy in KollaDiba Health Center.

## 2. Methods

### 2.1. Study Design and Period

Institution based cross-sectional study was carried out from April 15 to June 10, 2016 GC.

### 2.2. Study Area

The study was conducted at KollaDiba Health Center that is found in Dembia district, which is located in north Gondar Zone, Amhara region, and it is found 729 km from Addis Ababa and 35 km from the ancient city of Gondar. Dembia is one of the districts of North Gondar Administrative Zone and is geographically flat land. The area covers 1270 km with a total population of about 263,000 and the population of the district is predominately Amhara, with Orthodox Christianity being the main religion. There are one health center, one government hospital, and two private clinics. Koladiba Health Center is the first health center in Ethiopia established in 1954 EC, in the regime of Emperor Haile Selassie in collaboration with WHO and USAID to control malaria epidemic, which occurred in Kolladiba town in Dembia district. Currently Koladiba Health Center provides outpatient treatment, family planning, maternal and child health service, and a wide variety of health services including ART, TB, and adolescent health service. In ART clinic 956 ART clients were registered and 566 clients were on follow-up currently and the rest of the clients were transferred out, died, and lost.

### 2.3. Source Population

The study populations are all people living with HIV/AIDS who are on ART in Koladiba Health Center.

### 2.4. Study Population

Study populations are PLWHA on ART who attended at the time of data collection in Kolladiba Health Center and those who fulfilled inclusion criteria.

### 2.5. Inclusion Criteria

- ART attendees above 18 years.
- ART attendees who have been on ART for at least 3 months.

### 2.6. Exclusion Criteria

- Those who are mentally ill.
- Those who are unable to communicate verbally.

### 2.7. Sample Size Determination

The sample size was calculated by using single population formula where p-value was taken from a research done in Addis Ababa which is 63.1% consistent condom use, 95% level of confidence, and 5 % marginal error [7].

(1) $$\begin{array}{c} N=\frac{{\left(Z_{a2}\right)}^{2}p\left(1-p\right)}{d^{2}}=358 \end{array}$$

where

**n= **the minimum sample size,

**Z= **the desired level of confidence interval 95% (1.96),

**P=** the proportion of consistent condom use among PLWHA who are on ART (63.1%) from Addis Ababa public hospitals,

**d=** margin of error 5% (0.05).

Finally after adding 10% nonresponse rates, the sample size became 394.

### 2.8. Sampling Technique

Simple random sampling technique (computer generated table of random) was used to select the study population.

### 2.9. Data Collection Method

A well-structured pretested and interview based questionnaire was used to collect appropriate data from those who are on ART follow-up.

### 2.10. Data Quality and Clearance

To ensure the quality of data, 10% of sample size of the questionnaires was pretested in Gondar University Hospital ART Clinic. In addition to this, the questionnaire was designed in English, translated to Amharic, and then back translated to English to verify content. The collected data was checked by investigators for completeness, accuracy, and consistency.

### 2.11. Data Processing and Analysis

After data collection, data were entered and analyzed using SPSS version 22. Descriptive statistics was computed and binary and multiple logistic regressions were also conducted to examine the effect of selected independent variables on consistent condom use.

### 2.12. Ethical Consideration

Ethical clearance was obtained from School of Nursing Ethical Review Committee (ERC), in University of Gondar before the research started. Respondents were informed about the objective and purpose of the study and a signed informed consent was obtained from each respondent. Moreover, all the study participants were informed that they have a full right to participate or decline participating in the study and all the study participants were assured of confidentiality. No identifiers of study participants were included in data collection forms.

## 3. Results

### 3.1. Sociodemographic Characteristics of PLWHA Who Are on ART in Kolladiba Health Center

A total of 358 ART clients were participated in the study with response rate of 91%. Among study participants 138 (38.5%) were in the age category of 35-44 years. About 216 (60.3%) of the participants were female while 142 (39.7%) were male. More than half of the respondents 209 (58.4%) came from urban area. Among the respondents 325 (90.8%) were Orthodox followers. Around 124 (34.6%) of the study participants were housewives followed by private employee 95 (26.5%). Half (185, 51.7%) of the respondents were married. From those participants included in the study, 146 (40.8%) were unable to read and write (Table 1).

### 3.2. ART Related Characteristics

Two hundred thirty (64.2%) of the respondents had received ART for more than three years. According to health care providers report of 358 study subjects, about 293 (81.8%) have had improved health status after initiation of ART and 289(81.0%) of the participants had knowledge that ART can reduce HIV transmission (Table 2).

### 3.3. Sexual Behavior and Condom Use of ART Clients

Most of the respondents were sexually active. About three-fourths (270, 75.4%) of the respondents had had sex in the last three months of which 179 (66.3%) had sex with their regular spouse/cohabit partner and 231 (85.6%) had used condom. Almost all sexually active respondents had sex with only one sexual partner in the last three months.

Among sexually active respondents, more than half of them 130 (55.8%) had used condom consistently and about 101 (44.2%) used condom inconsistently. 39 (14.4%) of sexually active participants had never used condom at all. Among those condom users 170 (73.0%) of the respondents had used condom in their last sexual intercourse.

The main reason for condom use was to prevent transmission of new viral strains and other STIs 117(68.0%) followed by to prevent pregnancy 47(27.3%).

Among consistent condom users 76 (58.5%) were females, 90 (69.2%) were urban dwellers, 116 (89.2%) improved their health status, 103 (79.2%) were regular spouse/cohabit partner, 100 (76.9%) were to prevent new viral strain and other STIs, 108 (83.1%) were initiated by mutual decision, 124 (95.4%) had disclosed their serostatus, 108 (83.1%) had children, and 84 (64.6%) of the respondents have no desire to have children in the future.

The main reasons for inconsistent use and nonuse of condom at all were their assumption that they are already infected 35 (35.7%), positive status of their sexual partner 30 (30.6%), reducing sexual pleasure 13 (13.3%), desire to have children 10 (10.3), and partner refusal 9 (9.2%).

Among sexually active respondents 183 (67.8%) have had discussion about condom use and safe sex. Most of the sexually active respondents' partner serostatus who had sex in the last three months was positive HIV status 189 (70.0%) followed by negative serostatus 47 (17.4%). Majority of the participants 235 (87.4%) disclosed their status to their sexual partners. About 289 (80.7%) of the respondents had children; among them 122 (42.2%) had one child. Of the respondents 126 (35.2%) desired to have children in the future (Table 3).

### 3.4. Bivariate Analysis

#### 3.4.1. Bivariate Analysis of Sociodemographic Factors with Consistent Condom Use

Study participants aged 45 and older were 0.406 times less likely to use condom consistently than those aged 18-24 years (COR=0.406, 95% CI 0.190, 0.866). Of the respondents females were 0.92 times less likely to use condom consistently than males (COR=0.92, 95% CI: 0.642,1.838). The odds of utilizing condom inconsistently among rural respondents were 1.95 times more likely than in urban respondents (COR=1.95, 95%CI: 1.141, 3.36). Also consistent condom use by divorced respondents was 0.178 times less likely than single respondents (COR 0.178, 95% CI: 0.061, 0.517) (Table 4).

#### 3.4.2. Bivariate Analysis of Sexual Behaviors of Respondents with Consistent Condom Use

The chance of consistent condom use among respondents who had regular boy or girl friend was 0.123 times less likely than respondents who had regular spouse/cohabit (COR=0.123, 95% CI: 0.034,0.451). The odds of consistent condom use among the respondents who use condom by pressured partner were 0.053 times less likely than who use condom to prevent pregnancy (COR=0.053, 95% CI: 0.010,0.288). The chance of consistent condom use initiated by respondent him/herself was 10.8 times and 30.4 times more likely than condom use initiated by sexual partner and mutual decision, respectively.

The respondents whose partner had positive HIV serostatus and unknown serostatus were 0.127 and 0.220 times less likely to use condom consistently than those who have negative serostatus of their partner, respectively.

The respondents who did not disclose their serostatus to their partner were 0.27 times less likely to use condom consistently than those disclosed their HIV serostatus to their partner (COR=0.274, 95% CI: 0.1O2, 0.735).

The odds of inconsistent condom use among respondents who had the desire to have children were 1.863 times more likely than who did not have desire to have children ( COR=1.863, 95% CI: 1.096,3.166) (Table 5).

#### 3.4.3. Multivariate Analysis of Consistent Condom Use with Important Variables

Those variables having p-value <0.02 in bivariate analysis were taken into account in this model. In multivariate analysis place of residence and condom use initiated by sexual partner during sexual contact have been found to have an association with consistent condom use.

Respondents in rural residence were 0.326 times less likely to use condom consistently than respondents in urban residence (AOR=0.326, 95% CI: 0.109, 0.973). Among the participants, the chance of consistent condom use initiated by sexual partner was 0.031 times less likely than condom use initiated by respondent him/herself (AOR=0.031,95% CI:0.005,0.186) (Table 6).

## 4. Discussion

Condom use is a critical component in a comprehensive and sustainable approach to the prevention of HIV and other sexually transmitted infections (STIs) and for preventing unintended pregnancies especially among people with HIV-positive clients. The maximum protective effect of condoms is achieved when their use is consistent rather than occasional. Inconsistent use/nonuse of condom by PLWHA on ART will lead to further worsening the HIV infection epidemic and reinfection with new drug resistant viral strains.

Among sexually active respondents, 130 (55.8%) had used condom consistently in the past three months. The main reasons that respondents reported for not using condoms consistently were the assumption that they are already infected 35 (35.7%), positive status of their sexual partner 30 (30.6%), their opinion that condom reduces sexual pleasure 13 (13.3%), desire to have children 10 (10.3%), and partner refusal 9 (9.2%).

The level of consistent condom use was in line with the study done in Mekelle [8] and Kenya [9], 55.7% and 57.4%, respectively, but the result is lower than in studies done in London, Guatemala, South Africa, Uganda, and Gondar (73.0%, 81.7%, 75.8%, 65.0%, and 78.9%, respectively). The differences might be due to low educational status of the rural population in our study area, their negligence and carelessness to use condom, and lack of condom at a time of causal sexual contact. And it might also be due to lack of professional's commitment to create awareness on PLWHA on ART to use condom consistently.

The important factors associated with consistent condom use in this study were respondent's residence and condom use initiator. Consistent condom use was found to be less likely among PLWHA on ART who were in rural (AOR=0.326 96% CI: 0.109, 0.973) residence than in urban residence. This is in line with a study done in Gondar in which it was found that ART clients in rural residence were less likely to use condom consistently [10]. This could be due to information gap between urban and rural areas, lack of awareness, inadequate information about condom use, and lower educational status of rural population.

Among the participants, the chance of consistent condom use initiated by sexual partner (AOR=0.031, 95% CI: 0.005, 0.186) was less likely than condom use initiated by respondent him/herself. To our knowledge, this has not been previously reported.

Unlike other African studies [4, 7, 9–12] that have reported participants with higher educational status more likely to use condoms consistently compared to lower educational status participants, our study did not show any significant association between educational status and consistent condom use.

The main reasons for not using condom consistently mentioned were the assumption that HIV infected individuals do not need condoms since they are already infected (35.7%), positive status of their sexual partner (30.6%), their opinion that condom reduces sexual pleasure (13.3%), desire to have children (10.3%), and partner refusal (9.2%). Among these main reasons the assumption that HIV infected individuals do not need condoms since they are already infected, partner refusal, and desire to have children were in line with a study done in Mekella [8] and Bahr Dar [13]. Assumption that HIV infected individuals do not need condoms since they are already infected is also in line with a study done in Gondar [10]

## 5. Conclusion

In this study, we found out that consistent condom utilization among HIV clients on ART was low (55.8%). Place of residence and condom use initiation during sexual contact were significantly associated with consistent condom use.

## Figures and Tables

**Table 1:** Demographic characteristics of HIV infected persons in condom use survey, KollaDiba health center, North West Ethiopia 2016 G.C.

Variable	Frequency	Percent
*Age(in years)*		
18-24	20	5.6
25-34	108	30.2
35-44	135	38.5
≥45	95	25.7
Total	358	100.0
*Sex*		
Male	142	39.7
Female	216	60.3
Total	358	100.0
*Residence*		
Rural	149	41.6
Urban	209	58.4
*Religion*		
Orthodox	325	90.8
Muslim	17	4.7
Protestant	16	4.5
Other	0	0
Total	358	100.0
*Occupation*		
Government employee	21	5.9
Private employee	95	26.5
House wife	124	34.6
Daily laborer	51	14.2
Other	67	18.7
*Marital status*		
Single	25	7.0
Married	185	51.7
Divorced	87	24.3
Widowed	61	17.0
Total	358	100.0
*Educational status*		
Write and read	51	14.2
Unable to write and read	146	40.8
Primary school	82	22.9
Secondary school	55	15.4
College and above	24	6.7
Total	358	100.0

**Table 2:** ART related characteristics of HIV infected persons in condom use survey, Kollidiba health center, North West, Ethiopia, 2016.

Variable	Frequency	Percent
*Duration of ART(in months)*		
3-12	28	7.8
12-36	100	27.9
>36	230	64.2
Total	358	100
*Health status after initiation of ART*		
Improved	293	81.8
Same	52	14.5
Worsened	13	3.6
Total	358	100
*Perception that ART can reduce HIV transmission*		
Yes	290	81.0
No	68	19.0
Total	358	100

**Table 3:** Sexual behavior and condom use of HIV infected persons in condom use survey, Kolladiba health center North West Ethiopia, 2016.

Variable	Frequency	Percent
*Had sex in the last 3 months*		
Yes	270	75.4
No	88	24.6
Total	358	100.0
*Relationship with this partner*		
Regular, spouse/husband	179	66.3
Regular, or cohabiting boy/girl friend	66	24.4
Casual partner	25	9.3
Total	270	100.0
*Condom use in the last 3 months*		
Yes	231	85.6
No	39	14.4
Total	270	100.0
*How often?*		
Always	130	55.8
Sometimes	101	44.2
Total	231	100.0
*Condom use for last sexual intercourse*		
Yes	170	73.0
No	63	27.0
Total	233	100.0
*Reason for condom use*		
To prevent pregnancy	47	27.3
To prevent HIV and STIs	117	68.0
Pressured by sexual partner	8	4.7
Total	172	100.0
*Initiator of condom use*		
He/her self	34	19.7
Partner	20	11.6
Mutual decision	119	68.8
Total	173	100.0
*Reason for not using condom*		
Partner refused	9	9.2
Positive status of partner	30	30.6
Reduce sexual pleasure	13	13.3
Fear to ask partner to use condom	1	1.0
Wanted to have child	10	10.3
Since I am already infected	35	35.7
Total	98	100.0
*Discussion about condom use with partner*		
Yes	183	67.8
No	87	32.2
Total	270	100.0
*Partner sero status with whom had sex in the last 3 month*		
Negative	47	17.4
Positive	189	70.0
Unknown	34	12.6
Total	270	100.0
*HIV sero status disclosure to partner in the last 3 months*		
Yes	235	87.4
No	34	12.6
Total	269	100.0
*Do you have children*		
Yes	289	80.7
No	69	19.3
Total	358	100.0
*Number of children*		
1	72	24.9
2	95	32.9
>3	122	42.2
Total	289	100.0
*Desire to have children*		
Yes	126	35.2
No	232	64.8
Total	358	100.0

**Table 4:** Bivariate analysis by socio-demographic factors with consistent condom of the respondents in Kollidiba health center, North West Ethiopia, 2016.

Variable	Frequency (%)		COR	95%(CI)	p-value
	Consistent	Inconsistent			
	condom use	condom use			
*Age*					
18-24	6(46.2%)	7(53.8%)	1		
25-34	48(52.7%)	43(47.3%)	0.96	0.277,3.358	
35-44	57(67.1%)	28(32.9%)	0.74	0.355,1.542	
>45	19(45.2%)	23(54.8%)	0.41	*∗*(0.190,0.866)	0.02
*Sex*					
Male	54(55.1%)	44(44.9%)	1		
Female	76(57.1%)	57(42.9%)	0.92	(0.642,1.838)	0.10
*Residence*					
Rural	40(46.00%)	47(54.00)	1.95	*∗*(1.141,3.36)	0.015
Urban	90(62.5%)	54(37.5%)	1		
*Occupation*					
Government	13(72.2%)	5(27.8%)	1		
emp	29(48.3%)	31(51.7%)	0.39	(0.113,1.304)	0.13
Private emp	46(60.5%)	30(39.5%)	1.07	(0.468,2.443)	0.87
House wife	24(58.5%)	17(41.5%)	0.65	(0.293,1.450)	0.65
Daily laborer					
	18(50%)	18(50%)	O.71	(0.288,1.745)	0.45
Others					
*Marital status*					
Single	8(42.1%)	11((57.9%)	1		
Married	101(65.2%)	54(34.8%)	0.46	0.117,1.789	0.26
Divorced	16(43.2%)	21(56.8%)	0.18	*∗*(0.061,0.517)	0.010
Windowed	5(25%)	15(75%)	0.44	(0.131,1.457)	0.178
*Educational status*					
Write and read	17(58.6)	12(41.4%)	1.31	(0.403,4.263	0.65
Unable to	47(52.8%)	42(47.2%)	1.66	(0.605,4.551)	0.33
write/read	29(53.7%)	25(46.2%)	1.60	(0.553,4.636)	0.39
Primary school					
	24(61.5%)	15(38.5%)	1.16	(0.378, 3.567)	0.79
Secondary school					
College and	13(65%)	7(35%)	1		
above					
*Respondents health status*					
Improved	116(57.7%)	85(42.3%)	1		
Same	12(48.0%)	13(52.0%)	0.73	(0.101,5.306)	0.76
Worsened	2(50.0%)	2(50.0%)	1.08	(0.131,8.946)	0.94

**∗**COR=statistically significant (p<0.05)

**Table 5:** Bivariate analysis of sexual behaviors of respondents with consistent condom use in Kollidiba health center, North West Ethiopia 2016.

Variables	Frequency (%)		COR	95% CI	P- value
	Consistent	Inconsistent			
	condom use	condom use			
*Relationship of the sexual partner*					
Regular	103(65.2%)	55(34.8%)	1		
spouse/cohabit					
Regular boy/girl	24(42.1%)	33(57.9%)	0.12	*∗*(0.034,0.451)	0.00
friend					
Casual contact	3(18.8%)	138(1.2%)	0.32	*∗*(0.081,1.238)	0.09
*Reason for condom use*					
To prevent	28(60.9%)	18(39.1%)	1		
pregnancy					
					12
To prevent re	100(86.2%)	16(13.8%)	0.21	*∗*(0.039,1.180)	0.08
infection					
Pressured by	2(25%)	6(75%)	0.05	*∗*(0.010,0.288)	0.01
partner					
*Condom use Initiation by*					
Him/her self	17(50%)	17(50%)	1		
Partner	108(91.5%)	10(8.5%)	10.8	*∗*(4.246,27.4)	0.00
Mutual decision	5(26.3%)	14(73.7%)	30.24	*∗*(9.024,101.3)	0.00
*Discussion on condom use*					
Yes	122(68.9%)	55(31.1%)	1		
No	8(14.8%)	46(85.2%)	0.08	*∗*(0.035,0.173)	0.00
*HIV sero status of the partner*					
Negative	30(69.8%)	13(30.2%)	1		
Positive	95(57.2%)	71(42.8%)	0.13	(0.039,0.419)	0.00
Unknown	5(22.7%)	17(77.3)	0.22	(0.077,0.624)	0.00
*Disclosure of HIV status*					
Yes	124(59.3)	85(40.7)			
No	6(28.5%)	15(71.5%)	0.27	*∗*(0.102,0.735)	0.01
*Desire to have children*					
Yes	46(47.4%)	51(52.6%)	1.86	*∗*(1.096,3.166)	0.02
No	84(62.7%)	50(37.3%)	1		

*∗*COR=statistically significant (p<0.05)

**Table 6:** Multivariate analysis of predictors of consistent condom use in HIV infected persons, condom use survey, Kolladiaba health center, mNorth West Ethiopia, 2016.

Variables	Frequency		COR95% CI	AOR 95%
	Consistent	Inconsistent		
	condom	condom		
	use	use		
*Age*				
18-24	6(46.2%)	7(53.8%)	1	
25-34	48(52.7%)	43(47.3%)	0.964(0.277,3.358)	0.267(0.021,3.475)
35-44	57(67.1%)	28(32.9%)	0.740(0.355,1.542)	0.335(0.710,1.572)
>45	19(45.2%)	23(54.8%)	0.406(0.190,0.866)	0.247(0.570,1.080)
*Marital status*				
Single	8(42.1%)	11((57.9%)	1	
Married	101(65.2%)	54(34.8%)		
Divorced	16(43.2%)	21(56.8%)	0.178(0.061,0.517)	0.231(0.0192.854)
Windowed	5(25%)	15(75%)	0.438(0.131,1.457)	0.258(0.018,3.630)
*Residence*				
Rural	40(46.00%)	47(54.00)	1.95(1.141,3.36)	*∗∗*0.326(0.109,0.973)
Urban	90(62.5%)	54(37.5%)	1	
*Relationship of the sexual partner*				
Regular	103(65.2%)	55(34.8%)	1	
spouse/cohabit				
Regular	24(42.1%)	33(57.9%)	0.123(0.034,0.451)	0.860(0.013,55.880)
boy/girl friend				
Casual contact	3(18.8%)	1381.2(%)	0.317(0.081,1.238)	1.414(0.054,36.720)
*Reason for condom use*				
To prevent	28(60.9%)	18(39.1%)	1	
pregnancy				
To prevent	100(86.2%)	16(13.8%)	0.214(0.039,1.1180)	7.061(0.483,103.305)
reinfection				
Pressurized by	2(25%)	6(75%)	0.053(0.010,0.288)	1.817(0.148,22.269)
partner				
*Condom use Initiation by*				
Her/him self	17(50%)	17(50%)	1	
Partner	108(91.5%)	10(8.5%)	10.8(4.246,27.4)	*∗∗*0.031(0.005,0.186)
Mutual	5(26.3%)	14(73.7%)	30.24(9.024,101.3)	
decision				
*Discussion on condom use*				
Yes	122(68.9%)	55(31.1%)	1	
No	8(14.8%)	46(85.2%)	0.078(0.035,0.173)	0.355(0.066,1.913)
*HIV sero status of the partner*				
Negative	30(69.8%)	13(30.2%)	1	3.093(0.094,102.193)
Positive	95(57.2%)	71(42.8%)	0.127(0.039,0.419	
Unknown	5(22.7%)	17(77.3)	0.220(0.077,0.624)	0.878(0.033,23.397)
*Disclosure of HIV status*				
Yes	124(59.3)	85(40.7)	1	
No	6(28.5%)	15(71.5%)	0.274(0.102,0.735)	0.190(0.020,1.825)
*Desire to have children*				
Yes	46(47.4%)	51(52.6%)	1.863(1.096,3.166)	1.817(0.539,6.128)
No	84(62.7%)	50(37.3%)	1	

*∗∗* AOR= shows significant association P<0.05

## Data Availability

The data used to support the findings of this study are included within the article.

## Abbreviations

AIDS: Acquired Immune Deficiency Syndrome
ART: Antiretroviral Therapy
AOR: Adjusted odd ratio
COR: Crude odd ratio
HAART: Highly active antiretroviral therapy
HIV: Human Immune Deficiency Virus
PLWHA: People living with HIV/AIDS
SPSS: Statistical package for the social science
STI: Sexually transmitted infections
UNAIDS: Joint United Nations Program on HIV/AID
WHO: World Health Organization.
