# Corrigendum to “Consistent Condom Use and Associated Factors Among HIV-Positive Clients on Antiretroviral Therapy in North West Ethiopian Health Center, 2016 GC”

**DOI:** 10.1155/2019/8218107

**Published:** 2019-10-15

**Authors:** Mohammed Seid Ali, Eleni Tesfaye Tegegne, Mekibib Kassa Tessema, Kaleab Tesfaye Tegegne

**Affiliations:** ^1^School of Nursing, College of Medicine and Health Sciences, University of Gondar, Gondar, Ethiopia; ^2^Institute of Tropical Medicine (ITM), in Collaboration with University of Gondar, Gondar, Ethiopia; ^3^Department of Public Health, Hawassa Health Science College, Hawassa, Ethiopia

In the article titled “Consistent Condom Use and Associated Factors among HIV-Positive Clients on Antiretroviral Therapy in North West Ethiopian Health Center, 2016 GC” [1] the first affiliation is corrected as “School of Nursing, College of Medicine and Health Sciences, University of Gondar, Gondar, Ethiopia” due to a university's requirement. Additionally, the last name of the third author was given incorrectly as Tesemma. The author's name should have been written as Tessema. The corrected authors' list and affiliations are shown above.
